# Pharmacovigilance assessment of ropivacaine safety using FAERS data (2004–2024)

**DOI:** 10.1097/MD.0000000000046145

**Published:** 2026-05-12

**Authors:** Congcong Lai, Zengguang Bao, Zenan Chen, Ruichun Wang

**Affiliations:** a Department of Anesthesiology, Xiangshan Hospital of TCM Medical and Health Group, Ningbo, China; b Department of Anesthesiology, Ningbo No. 2 Hospital, Ningbo, China.

**Keywords:** adverse drug events, disproportionality analysis, FAERS, pharmacovigilance, ropivacaine

## Abstract

Despite widespread clinical use of the long-acting local anesthetic ropivacaine, its population-level safety remains under-explored. This study aimed to analyze and categorize adverse events (AE) associated with ropivacaine reported in the US Food and Drug Administration Adverse Event Reporting System (FAERS). We retrospectively extracted reports on adverse drug events (ADEs) from the FAERS database from the first quarter of 2004 to the fourth quarter of 2024. Using disproportionality analysis, including the reporting odds ratio, proportional reporting ratio, Bayesian confidence propagation neural network, and the empirical Bayesian geometric mean, we assessed whether there was a significant association between ropivacaine and ADEs. The Weibull shape parameter was used to test the time to onset curve. The Kaplan–Meier method was employed to evaluate the cumulative incidence. In the FAERS database, ropivacaine was identified as the primary suspect in 1463 AE reports. The adverse reactions induced by ropivacaine involved 3 system organ categories and 21 high-level group terms. At the preferred term level, 181 positive ADEs were detected. Common ADEs included complications of anesthesia, Horner’s syndrome, delayed awakening after anesthesia, systemic toxicity of local anesthetics, and monoparesis. Gender differences existed in ADEs related to ropivacaine. The median time to onset of ADEs related to ropivacaine was 2 days (range: 1–3 days). Weibull shape parameter testing revealed an early failure pattern for ropivacaine-associated ADEs. This pharmacovigilance study identifies significant safety signals associated with ropivacaine, particularly neurological and cardiac events that predominantly occur within the first 72 hours after administration. The detection of potential gender-specific reporting patterns and specific high-strength signals warrants further clinical investigation. These findings provide valuable hypothesis-generating evidence from real-world data to guide targeted monitoring and inform future research on ropivacaine’s safety profile.

## 1. Introduction

Ropivacaine is a long-acting local anesthetic that works by blocking sodium channels in nerve cells, preventing them from sending pain signals.^[1,2]^ Its key clinical advantage is its ability to preferentially block pain-transmitting fibers while largely sparing motor fibers.^[3]^ This selective blockade, coupled with its potent analgesic effect, has rendered ropivacaine the drug of choice in various clinical applications, including surgical anesthesia,^[4]^ postoperative pain management,^[5]^ and labor analgesia.^[6]^

Although ropivacaine has demonstrated significant advantages in clinical applications, its safety issues remain a focal point of ongoing concern in the medical community. Local anesthetic systemic toxicity is one of the most common and serious complications associated with the use of ropivacaine, with symptoms including arrhythmias, neurological complications, and other systemic toxic reactions.^[7]^ Arrhythmias result from the inhibition of sodium channels by the local anesthetic, which affects the electrical activity of the myocardium and may lead to arrhythmias or even cardiac arrest.^[8]^ Additionally, ropivacaine may cause various degrees of neurological complications, such as drowsiness, sensory loss, and tonic-clonic seizures.^[9]^ While some case reports exist, a comprehensive analysis of ropivacaine’s ADEs is still lacking, and further exploration of this issue is crucial for clinical decision-making and ensuring patient safety.^[10,11]^

The US Food and Drug Administration (FDA) Adverse Event Reporting System (FAERS) is an authoritative and continuously updated drug safety database that provides valuable resources for pharmacovigilance and risk assessment.^[12–14]^ The present study aimed to systematically analyze the ADEs associated with ropivacaine since its market approval using the FAERS database. These findings contribute to a better understanding of the safety profile of ropivacaine and facilitate its safe and effective use in clinical anesthesia.

## 2. Materials and methods

### 2.1. Data sources

In this study, we conducted a retrospective safety analysis of individuals using ropivacaine, utilizing the FAERS database, covering data from the first quarter of 2004 to the fourth quarter of 2024. All data were officially downloaded from the FDA in March 2025. The data collected in FAERS is organized into 7 tables: patient demographics (DEMO), drug information (DRUG), medication administration indications, date of treatment initiation and end date of reported medication, adverse drug reaction information, patient outcome information, and reporting source information. These tables are linked by a unique identifier, PRIMARYID, within the FAERS database structure. Due to the anonymous coding system employed by FAERS, this study was exempt from institutional review board approval.

### 2.2. Data extraction and processing

The FDA-recommended method was employed to remove duplicate reports. We organized the reports by case identifiers (CASEIDs), FDA receipt date (FDA_DT), and primary identifiers (PRIMARYID). For reports with the same CASEID, the 1 with the latest FDA_DT value was retained. For reports with identical CASEID and FDA_DT, the report with the highest PRIMARYID value was retained. This method allowed for the identification and removal of duplicate reports.

**Figure 1.**
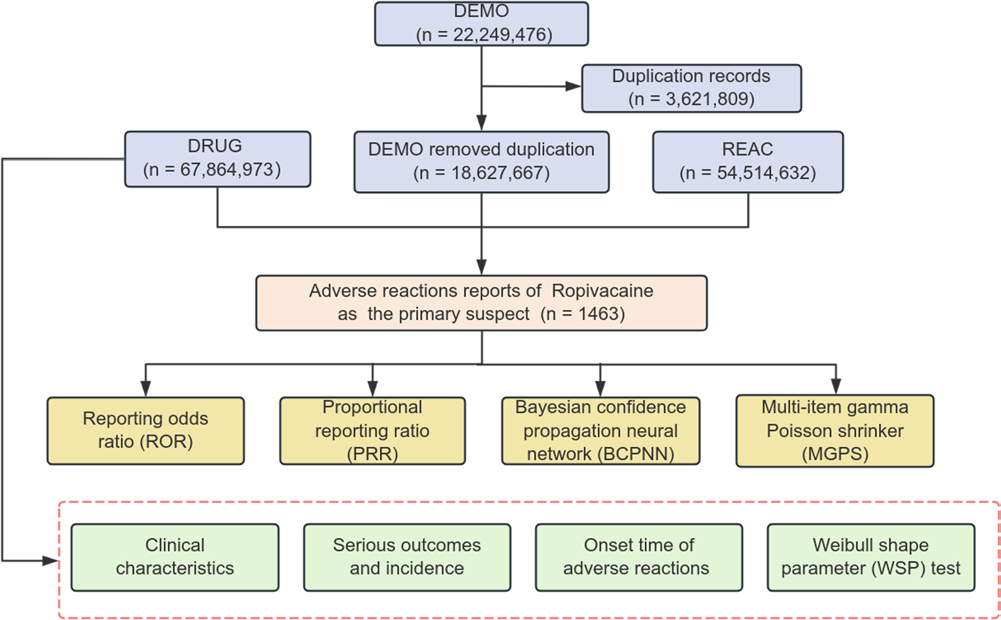
Flowchart of the entire study. BCPNN = Bayesian confidence propagation neural network, DEMO = demographics, DRUG = drug information, MGPS = multi-item gamma poisson shrinker, REAC = drug reaction information, ROR = reporting odds ratio, PRR = proportional reporting ratio, WSP = Weibull shape parameter.

In this study, the generic and brand names of ropivacaine were identified using the MeSH browser. The brand names “Naropin” and “Naropeine,” as well as the generic name “Ropivacaine,” were used to search the FAERS database. Only ADEs where ropivacaine was the primary suspect drug were included in this analysis. Clinical characteristics related to ropivacaine-associated ADEs were collected, including gender, age, reporting region, reporter type, drug usage duration, and ADEs onset time. ADEs related to ropivacaine were classified using the Medical Dictionary for Regulatory Activities (MedDRA) into system organ cases (SOC), high-level group terminology (HLGT), and preferred term categories. Figure 1 illustrates the entire analysis process.

### 2.3. Time to onset (TTO) analysis

The interval between the occurrence time of ADEs (EVENT_DT, DEMO file) and the start time of ropivacaine usage (START_DT, THER file) was used to estimate the time to onset (TTO). In the integration of the median, quartiles, minimum, maximum, and Weibull shape parameter (WSP) evaluations for TTO, data issues such as EVENT_DTSTART_DT, incorrect dates, and missing data were excluded to ensure the accuracy of the study. The WSP analysis uses the scale (α) and shape (β) parameters to assess the occurrence of ADEs over time, with these parameters determining the amplitude and structure of the distribution function. β value < 1 (95% CI < 1) indicates a decrease in risk over time (early failure type), β value equal to or near 1 (95% CI includes 1) indicates a constant risk (random failure type), and β > 1 (95% CI excludes 1) indicates an increase in risk (wear-out failure type). The Kaplan–Meier plot shows the cumulative incidence of ADEs, and the log-rank test compares the groups. Statistical significance was defined as *P* < .05. This analysis of time to onset (TTO) has an important limitation. Among all 1463 cases, only 127 (8.7%) contained reliable onset dates. Consequently, the subsequent descriptive statistics of TTO (e.g., median, interquartile range) are based solely on this subset of cases. Their representativeness of the entire case population may be limited, and extrapolation to the broader population requires extreme caution.

### 2.4. Statistical analysis

In our study, we employed disproportionate analysis, a commonly used method in pharmacovigilance research, to identify potential signals between the drug and ADEs. This widely adopted data mining approach evaluates the correlation between the drug and ADEs by comparing the observed frequencies in exposed and nonexposed populations through 2 × 2 contingency tables (as shown in Table S1, Supplemental Digital content, https://links.lww.com/MD/Q808). We used the reporting odds ratio, proportional reporting ratio (PRR), Bayesian confidence propagation neural network (BCPNN), and empirical Bayesian geometric mean (EBGM) for signal detection. ROR and PRR are frequency-based methods that are known to have high sensitivity but low specificity. BCPNN and EBGM are Bayesian algorithms that can handle complex variables but have lower sensitivity in signal detection. We applied all 4 algorithms to ensure the stability and reliability of the study results. In this study, an adverse event (AE) term was considered significant only if it met the threshold criteria of all 4 algorithms.

Disproportionate positive signals in this study were defined based on the following criteria: at least 3 reported cases, the lower limit of the 95% confidence interval ROR was >1, PRR at least 2, a chi-square value (χ²) of at least 4, IC025 > 0, and EBGM > 2^[15]^ (Table S2, Supplemental Digital content, https://links.lww.com/MD/Q808). The higher the values of ROR, PRR, BCPNN, and EBGM, the stronger the ADE signals, indicating a stronger statistical relationship between the target drug and the target ADEs. To enhance the reliability of the study results, a separate disproportionate analysis was conducted, stratified by patient gender (male, female). All statistical analyses in this study were performed using R software (version 4.3.2; Vienna, Austria). The analysis was primarily conducted using the easy FAERS, survival, and fitdistrplus packages.

## 3. Results

### 3.1. Basic information on reports of ADEs related to ropivacaine

Among all AEs, females (51.13%) accounted for a higher proportion than males (30.35%), with gender information missing in 18.52% of reports (Table 1). The majority of cases (41.35%) occurred in the 18 to 64 age group, while age data were missing for 34.59% of reports. Regarding weight distribution, a substantial portion of the data (69.38%) lacked weight information, which limited a more in-depth analysis of the relationship between weight and AEs. Among reports with specific weight data, the most common weight group was 50 to 100 kg (23.86%), followed by individuals weighing <50 kg (3.96%). Healthcare professionals (including physicians, pharmacists, and other healthcare providers) submitted 90.70% of the reports, with reporter information missing in 4.58% of cases. The top 5 countries reporting ropivacaine-related AEs were the United States (36.30%), France (15.65%), Japan (8.00%), Portugal (7.18%), and Australia (3.55%). Regarding serious outcomes associated with ropivacaine, other serious outcomes (31.72%) were reported most frequently, followed by hospitalization (21.39%) and life-threatening events (14.56%), with outcome information missing in 18.87% of reports.

**Table 1:** Basic information on ropivacaine-related ADEs in the FAERS database (Q1 2004 to Q4 2024).

Characteristics	Ropivacaine
N	1463
Sex	
F	748 (51.13%)
M	444 (30.35%)
Missing	271 (18.52%)
Weight	
<50 kg	58 (3.96%)
>100 kg	41 (2.80%)
50–100 kg	349 (23.86%)
Missing	1015 (69.38%)
Age	
<18	67 (4.58%)
>85	12 (0.82%)
18–64	605 (41.35%)
65–85	273 (18.66%)
Missing	506 (34.59%)
Reported person	
Consumer (CN)	54 (3.69%)
Health professional (HP)	175 (11.96%)
Lawyer (LW)	12 (0.82%)
Physician (MD)	495 (33.83%)
Other health professional (OT)	397 (27.14%)
Pharmacist (PH)	260 (17.77%)
Registered Nurse (RN)	3 (0.21%)
Missing	67 (4.58%)
Reported countries (top 5)	
United states	531 (36.30%)
France	229 (15.65%)
Japan	117 (8.00%)
Portugal	105 (7.18%)
Australia	52 (3.55%)
Serious outcome	
Congenital anomaly (CA)	3 (0.21%)
Death (DE)	75 (5.13%)
Disability (DS)	87 (5.95%)
Hospitalization (HO)	313 (21.39%)
Life-threatening (LT)	213 (14.56%)
Other serious outcomes (OT)	464 (31.72%)
Required intervention (RI)	32 (2.19%)
Missing	276 (18.87%)

CA = congenital anomaly, CN = consumer, DE = death, DS = disability, HO = hospitalization, HP = health professional, LT = life-threatening, LW = lawyer, MD = doctor of medicine, N = case numbers, OT = other serious outcomes, PH = pharmacist, RI = required intervention, RN = registered nurse.

### 3.2. Signal mining of ropivacaine-related ADEs

#### 3.2.1. Signal detection at the SOC level

Ropivacaine-related ADEs were identified as potential signals using ROR, PRR, BCPNN, and MGPS analyses. At the SOC level, ropivacaine-related ADEs primarily involved 3 SOCs. The 3 affected systems are: various neurological disorders (n = 888, ROR = 3.23, PRR = 2.72, IC = 1.44, EBGM = 2.72), cardiovascular diseases (n = 390, ROR = 4.14, PRR = 3.82, IC = 1.93, EBGM = 3.82), and immune system disorders (n = 104, ROR = 2.46, PRR = 2.42, IC = 1.27, EBGM = 2.42; Table 2).

**Table 2:** Signal detection at the system organ class level.

System organ class (SOC)	N	ROR (95%Cl)	PRR (χ²)	EBGM (EBGM05)	IC (IC025)
Cardiac disorders	390	4.14 (3.73–4.6)	3.82 (835.39)	3.82 (3.50)	1.93 (1.78)
Nervous system disorders	888	3.23 (3.00–3.49)	2.72 (1053.28)	2.72 (2.55)	1.44 (1.33)
Immune system disorders	104	2.46 (2.02–2.98)	2.42 (87.31)	2.42 (2.05)	1.27 (0.99)

Only SOCs meeting the significance criteria for all 4 signal detection algorithms are reported. Other SOCs were screened but did not reach the signal thresholds.

CI = confidence interval, EBGM05 = empirical Bayes geometric mean 5th percentile, EBGM = empirical Bayesian geometric mean, IC = information component, IC025 = information component 2.5th percentile, N =case numbers, PRR = proportional reporting ratio, ROR = reporting odds ratio, SOC = system organ class, χ^2^ = chi-squared.

#### 3.2.2. Signal detection at the high-level group term level

Ropivacaine-related ADEs were identified as potential signals using ROR, PRR, BCPNN, and MGPS analyses. At the HLGT level, ropivacaine was associated with 21 categories. Table 3 displays the number of cases occurring in different HLGTs, ranked by the number of ADEs in each HLGT. The top 3 most common categories are: neurological disorders (n = 398, ROR = 2.96, PRR = 2.76, EBGM = 2.76, CI = 1.46), arrhythmias (n = 325, ROR = 9.29, PRR = 8.59, EBGM = 8.58, CI = 3.1), and movement disorders (n = 176, ROR = 5.69, PRR = 5.48, EBGM = 5.48, CI = 2.45).

**Table 3:** Signal detection at the high-level group term (HLGT) level.

HLGT	N	ROR (95%Cl)	PRR (χ²)	EBGM (EBGM05)	IC (IC025)
Neurological disorders nec	398	2.96 (2.67–3.29)	2.76 (463.60)	2.76 (2.53)	1.46 (1.31)
Cardiac arrhythmias	325	9.29 (8.29–10.41)	8.59 (2198.41)	8.58 (7.80)	3.1 (2.93)
Movement disorders (including parkinsonism)	176	5.69 (4.89–6.62)	5.48 (649.27)	5.48 (4.82)	2.45 (2.23)
Procedural related injuries and complications nec	173	10.97 (9.41–12.77)	10.52 (1495.10)	10.51 (9.25)	3.39 (3.17)
Exposures, chemical injuries and poisoning	153	5.15 (4.38–6.05)	4.98 (490.63)	4.98 (4.35)	2.32 (2.08)
Decreased and nonspecific blood pressure disorders and shock	114	5.91 (4.90–7.12)	5.76 (450.53)	5.76 (4.93)	2.53 (2.25)
Allergic conditions	103	3.13 (2.57–3.81)	3.07 (145.24)	3.07 (2.61)	1.62 (1.33)
Seizures (including subtypes)	91	7.39 (6.00–9.1)	7.24 (490.78)	7.24 (6.08)	2.86 (2.55)
Neuromuscular disorders	59	10.69 (8.26–13.82)	10.54 (509.74)	10.53 (8.49)	3.4 (3.02)
Peripheral neuropathies	41	4 (2.94–5.44)	3.97 (91.13)	3.96 (3.06)	1.99 (1.54)
Spinal cord and nerve root disorders	37	18.17 (13.14–25.13)	18.01 (593.91)	17.99 (13.72)	4.17 (3.70)
Ocular neuromuscular disorders	27	7.17 (4.91–10.47)	7.13 (142.36)	7.13 (5.19)	2.83 (2.29)
Thoracic disorders (excluding lung and pleura)	20	55.23 (35.56–85.79)	54.95 (1055.39)	54.74 (37.87)	5.77 (5.14)
Cranial nerve disorders (excluding neoplasms)	16	4.19 (2.56–6.84)	4.17 (38.63)	4.17 (2.77)	2.06 (1.36)
Retina, choroid and vitreous hemorrhages and vascular disorders	12	5.95 (3.38–10.49)	5.94 (49.27)	5.93 (3.69)	2.57 (1.77)
Obstetric and gynaecological therapeutic procedures	11	4.63 (2.56–8.37)	4.62 (31.21)	4.62 (2.81)	2.21 (1.37)
Central nervous system infections and inflammations	9	22.55 (11.72–43.39)	22.5 (184.59)	22.46 (12.99)	4.49 (3.57)
Gastrointestinal investigations	7	3.82 (1.82–8.01)	3.81 (14.51)	3.81 (2.05)	1.93 (0.91)
Neonatal respiratory disorders	4	4.74 (1.78–12.64)	4.74 (11.79)	4.74 (2.08)	2.24 (0.95)
Nervous system neoplasms benign	3	10.81 (3.48–33.56)	10.81 (26.68)	10.8 (4.19)	3.43 (1.99)
Ancillary infectious topics	3	7.87 (2.54–24.42)	7.87 (17.97)	7.86 (3.05)	2.97 (1.53)

Extremely high ROR values (e.g., >1000) are typically associated with rare events and small numbers of case reports, and should therefore be interpreted with caution.

CI = confidence interval, EBGM05 = empirical Bayes geometric mean 5th percentile, EBGM = empirical Bayesian geometric mean, HLGT = high-level group terminology, IC = information component, IC025 = information component 2.5th percentile, N = case numbers, OR = reporting odds ratio, PRR = proportional reporting ratio, ROR = reporting odds ratio, χ^2^ = chi-squared.

#### 3.2.3. Signal detection at the preferred term level

Our examination of PT signals identified 181 significant PTs that met the criteria of all 4 algorithms. These were ranked using the ROR algorithm, with the PTs listed in Tables 4 and S3, Supplemental Digital content, https://links.lww.com/MD/Q808. After ranking by ROR, the top 5 ADEs with the highest case numbers are: complications of anesthetics (n = 50, ROR = 298.05, PRR = 294.18, EBGM = 288.21, CI = 8.17), Horner’s syndrome (n = 32, ROR = 1016.94, PRR = 1008.46, EBGM = 941.43, CI = 9.88), delayed awakening after anesthesia (n = 26, ROR = 458.42, PRR = 455.32, EBGM = 441.16, CI = 8.79), systemic toxicity of local anesthetics (n = 18, ROR = 454.526, PRR = 452.4, EBGM = 438.41, CI = 8.78), and monoplegia (n = 18, ROR = 64.18, PRR = 63.88, EBGM = 63.60, CI = 5.99).

**Table 4:** Top 20 signal detection based on PT level ranked by ROR.

PTs	N	ROR (95%Cl)	PRR (χ²)	EBGM (EBGM05)	IC (IC025)
Spinal anesthesia	6	1269.14 (550.19–2927.56)	1267.16 (6967.04)	1163.09 (577.94)	10.18 (9.04)
Therapeutic product effect prolonged	23	1029.59 (673.52–1573.91)	1023.42 (21,908.30)	954.46 (669.17)	9.9 (9.29)
Horner’s syndrome	32	1016.94 (709.48–1457.64)	1008.46 (30,064.28)	941.43 (696.56)	9.88 (9.36)
Harlequin syndrome	4	994.01 (360.49–2740.92)	992.98 (3703.99)	927.93 (397.13)	9.86 (8.52)
Macular ischemia	7	848.12 (395.33–1819.53)	846.58 (5578.31)	798.84 (421.78)	9.64 (8.59)
Phrenic nerve paralysis	11	843.76 (458.87–1551.5)	841.35 (8714.68)	794.18 (477.07)	9.63 (8.77)
Nerve root injury	4	798.01 (291.31–2186.04)	797.18 (3011.08)	754.72 (324.78)	9.56 (8.23)
Femoral nerve injury	3	772.42 (241.54–2470.1)	771.81 (2189.99)	731.94 (276.72)	9.52 (8.02)
Anterior spinal artery syndrome	4	745.51 (272.63–2038.61)	744.73 (2822.39)	707.55 (304.93)	9.47 (8.14)
Maternal exposure during delivery	31	650.32 (453.10–933.39)	645.07 (19,066.27)	616.99 (456.00)	9.27 (8.75)
Delayed recovery from anesthesia	26	458.42 (309.81–678.31)	455.32 (11,419.09)	441.16 (317.84)	8.79 (8.22)
Local anesthetic systemic toxicity	18	454.52 (283.97–727.51)	452.4 (7856.07)	438.41 (295.77)	8.78 (8.10)
Diaphragmatic paralysis	14	373.03 (219.21–634.77)	371.67 (5042.97)	362.18 (232.14)	8.5 (7.74)
Epidural hemorrhage	3	363.1 (115.38–1142.69)	362.82 (1055.40)	353.77 (135.55)	8.47 (7.00)
Neuromuscular block prolonged	8	334.62 (165.87–675.06)	333.92 (2594.19)	326.25 (181.35)	8.35 (7.37)
Spinal cord ischemia	3	303.45 (96.65–952.72)	303.21 (884.69)	296.87 (113.98)	8.21 (6.75)
Anaesthetic complication	50	298.05 (224.84–395.09)	294.18 (14,312.13)	288.21 (227.66)	8.17 (7.76)
Cauda equina syndrome	12	251.6 (142.05–445.63)	250.81 (2933.81)	246.46 (152.76)	7.95 (7.14)
Suspected product quality issue	21	228.25 (148.14–351.68)	227 (4650.68)	223.44 (155.62)	7.8 (7.18)
Neuromuscular blockade	3	216.75 (69.27–678.18)	216.58 (634.05)	213.33 (82.14)	7.74 (6.28)

Extremely high ROR values (e.g., >1000) are typically associated with rare events and small numbers of case reports, and should therefore be interpreted with caution.

CI = confidence interval, EBGM05 = empirical Bayes geometric mean 5th percentile, EBGM = empirical Bayesian geometric mean, IC = information component, IC025 = information component 2.5th percentile, N = case numbers, OR = reporting odds ratio, PRR = proportional reporting ratio, PT = preferred term, ROR = reporting odds ratio, χ^2^ = chi-squared.

### 3.3. Subgroup analysis

The subgroup analysis revealed that in females, the most frequently reported ADEs ranked by ROR were complications of anesthetics (n = 32, ROR = 351.15, PRR = 345.61, EBGM = 337.90, CI = 8.40), Horner’s syndrome (n = 28, ROR = 1721.69, PRR = 1697.88, EBGM = 1526.36, CI = 10.58), neurotoxicity (n = 20, ROR = 1721.69, PRR = 1697.88, EBGM = 1526.36, CI = 10.58), anaphylactic shock (n = 20, ROR = 54.83, PRR = 54.29, EBGM = 54.1, CI = 5.76), and generalized tonic-clonic seizures (n = 20, ROR = 43.87, PRR = 43.44, EBGM = 43.32, CI = 5.44; Table S4, Supplemental Digital content, https://links.lww.com/MD/Q808). In males, the most frequently reported ADEs were anaphylactic shock (n = 18, ROR = 33.23, PRR = 32.78, EBGM = 32.71, CI = 5.03), complications of anesthetics (n = 16, ROR = 326.79, PRR = 322.75, EBGM = 315.60, CI = 8.30), ventricular fibrillation (n = 14, ROR = 38.60, PRR = 38.19, EBGM = 38.09, CI = 5.25), systemic toxicity of local anesthetics (n = 10, ROR = 935.40, PRR = 928.17, EBGM = 871.29, CI = 9.77), and sinus tachycardia (n = 10, ROR = 29.06, PRR = 28.85, EBGM = 28.79, CI = 4.85; Table S5, Supplemental Digital content, https://links.lww.com/MD/Q808).

### 3.4. Adverse event time to onset (TTO) analysis

**Figure 2.**
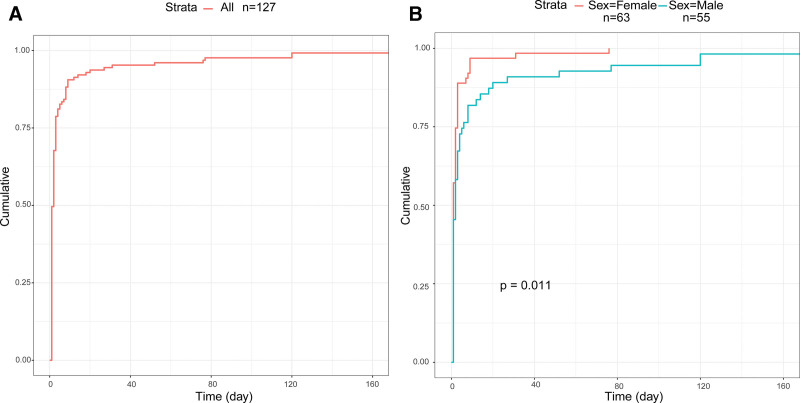
(A) Cumulative incidence of ADEs in the total patient population receiving ropivacaine treatment. (n = 127). (B) Cumulative incidence of ADEs in patients stratified by gender (female = 63, male = 55). ADE = adverse drug event.

After excluding reports with inaccurate, missing, or unknown onset times, a total of 127 reports were included, with the median TTO for ADEs being 2 days. To investigate factors associated with TTO, patients were stratified by gender. A total of 63 reports from females and 55 reports from males provided onset times. For females, the median TTO was 1 day (IQR 1–2.5), while for males, the median TTO was 2 days (IQR 1–5.5). Figure 2 shows the cumulative incidence of ADEs stratified by gender. There was a statistically significant difference in TTO between female and male patients, indicating that gender is a factor influencing the TTO of ADEs. Additionally, WSP analysis (Table 5) revealed that both the overall ropivacaine drug and the gender-stratified groups followed an early-decline pattern. This suggests that the incidence of ropivacaine-related ADEs decreases over time.

**Table 5:** Weibull shape parameter analysis.

	Time to onset (d)			Weibull distribution				Failure type
				Scale parameter		Shape parameter		
	N	Median (IQR)	Min–max	α	95% CI	β	95% CI	
Ropivacaine	127	2 (1–3)	1–169	4.46	3.15–5.76	0.63	0.56–0.70	Early failure
Female	63	1 (1–2.5)	1–76	6.78	3.46–10.10	0.58	0.47–0.68	Early failure
Male	55	2 (1–5.5)	1–169	2.95	1.96–3.94	0.78	0.66–0.90	Early failure

CI = confidence interval, IQR = interquartile range, N = case numbers.

## 4. Discussion

This study provides valuable insights into the characteristics of ADEs associated with ropivacaine, based on data from the FAERS database spanning from Q1 2004 to Q4 2024. Out of a total of 22,249,476 case reports, 1463 were related to ropivacaine, a widely used local anesthetic. The study analyzes these ADE reports and evaluates the performance of various intensity signals at multiple levels, including SOC, HLGT, and PT. The findings reveal a significant clustering of AEs in the nervous system, cardiovascular system, and immune-related systems, suggesting that ropivacaine use may be associated with a higher frequency of side effects in these areas. Additionally, the study identifies a higher incidence of serious AEs within the first 2 days after administration, highlighting the importance of early monitoring.

Compared to the study by Schweitzer-Chaput et al, which focused on pediatric local anesthetic systemic toxicity in a French database and emphasized dosing errors in infants, our analysis – spanning a broader age range – identified a high proportion of neurological and cardiac events even at recommended doses, underscoring the need for vigilant monitoring across all age groups.^[16]^ Our use of multiple disproportionality analyses and stringent criteria (requiring signals across 4 algorithms) enhances signal reliability, addressing concerns raised by Dagenais et al regarding case definition challenges in pharmacovigilance.^[11]^ While Contreras-Salinas et al highlighted mydriasis as an unexpected local anesthetics effect – a signal not prominent in our ropivacaine data – this may reflect underreporting or drug-specific profiles.^[17]^ Additionally, the gender-based differences in AE timing and type observed in our study, as also noted in recent FAERS analyses such as that by Liu et al regarding sugammadex, reinforce the importance of tailored, demographic-aware monitoring strategies and standardized toxicity definitions to improve clinical risk management.^[14]^

Our analysis identified a higher proportion of ropivacaine-associated ADEs reported in females compared to males. This observed disparity aligns with broader pharmacovigilance data, which often show a female skew in ADE reporting. The gender hypothesis proposed by Lee et al offers a valuable framework for interpreting such findings, suggesting that gendered social factors-such as differential healthcare utilization, reporting behaviors, and clinical interactions-may significantly influence these patterns, rather than purely biological sex differences.^[18]^ For instance, the prominent signals for neurological and cardiovascular events in both sexes are consistent with ropivacaine’s known pharmacology. However, the specific constellation of reported ADEs and the shorter median TTO in females could be influenced by several factors. These include the prevalent use of ropivacaine in obstetric anesthesia, a female-specific clinical context, and potential gender-based differences in symptom perception or reporting thresholds.^[6,19,20]^ The finding that a significant portion of reports lacked sex data (~19%) also necessitates cautious interpretation of these subgroup comparisons. It is critical to emphasize that these results, derived from disproportionality analysis, reflect reporting associations and not necessarily true differences in incidence or biological susceptibility. The disparity should not be directly attributed to sex-specific pharmacology without further confirmation from controlled studies that can account for confounding variables such as indication, dosing, and comorbidities. Future investigations incorporating gender-sensitive research designs are warranted to better elucidate the complex interplay of sex and gender in local anesthetic safety.

Using multiple analytical algorithms (ROR, PRR, BCPNN, MGPS), our analysis identified neurological, cardiovascular, and immune system disorders as the primary categories for ropivacaine-related ADEs. Notably, neurological disorders were the most prevalent (888 cases, ROR = 3.23), reinforcing its neurotoxic potential and necessitating vigilant postoperative neurological monitoring. Careful drug and dose selection is crucial for patients with preexisting neurological conditions or those undergoing high-risk surgeries.^[21]^ Cardiovascular events (390 cases, ROR = 4.14), particularly arrhythmias (325 cases, ROR = 9.29), underscore a significant cardiotoxic risk. To mitigate this, clinical practice should employ ultrasound guidance, incremental injection techniques, and have emergency equipment like lipid emulsion readily available.^[22–24]^ Immune system disorders (104 cases, ROR = 2.46), while less frequent, highlight the risk of hypersensitivity reactions, warranting enhanced allergy history screening and preparedness for delayed allergic responses. Overall, these findings emphasize the need for integrated risk assessment, vigilant monitoring, and tailored clinical strategies to minimize severe outcomes associated with ropivacaine. Future research should focus on elucidating the underlying toxicity mechanisms to establish safer clinical guidelines.

While this study provides a comprehensive post-market safety evaluation of ropivacaine using the largest real-world data sample to date, several critical limitations inherent to the FAERS database must be emphasized. First, significant underreporting and selective reporting bias are fundamental constraints of spontaneous reporting systems. The number of reports in FAERS reflects reporting rates rather than true incidence, and the tendency to report severe or novel AEs may disproportionately amplify certain signals while obscuring others. Second, extensive missing demographic and clinical data severely hinders a nuanced analysis. In our dataset, detailed weight was missing in 69.38% of reports, age was missing in 34.59%, and sex was absent in 18.52%. More importantly, the lack of critical clinical details – such as patient comorbidities, severity of underlying diseases, and concomitant medications – prevents adequate control for confounding factors, making it difficult to isolate the specific effect of ropivacaine. Third, the absence of definitive causality assessment is a key constraint. Disproportionality analysis is designed to detect statistical associations and generate safety signals; it does not quantify risks or establish a causal link between the drug and the reported events. The findings herein require confirmation through well-designed analytical epidemiological studies. Finally, due to the limited number of reports with documented event onset times, the findings from the TTO analysis should be interpreted with caution. Future prospective studies are needed to systematically collect such data for a more robust temporal assessment. Despite these substantial limitations, which are inherent to pharmacovigilance research using FAERS, the comprehensive characterization of ropivacaine-related ADEs presented here offers valuable insights for informing clinical practice and guiding further investigative research.

## 5. Conclusion

This large-scale FAERS pharmacovigilance study characterizes the spectrum, timing, and potential sex-specific differences in ropivacaine-associated AEs. Our findings suggest the need for vigilant clinical monitoring, particularly during the initial 72 hours following exposure. However, given the inherent limitations of spontaneous reporting data – including lack of denominators, potential underreporting, and confounding – these results should be interpreted as hypothesis-generating. The observed sex-specific patterns in reported ADEs warrant further investigation in controlled settings before any implications for dosing can be considered. Prospective studies and randomized trials are essential to confirm these signals, establish causality, and ultimately support evidence-based updates to ropivacaine safety guidance.

## Author contributions

**Conceptualization**: Congcong Lai, Ruichun Wang.

**Data curation**: Zengguang Bao.

**Formal analysis**: Zengguang Bao.

**Resources**: Congcong Lai.

**Validation**: Zenan Chen.

**Visualization**: Zenan Chen.

**Writing – original draft**: Congcong Lai.

**Writing – review & editing**: Ruichun Wang.

## Supplementary Material
