# Supplementary material for: Pharmacovigilance assessment of ropivacaine safety using FAERS data (2004–2024)
Source: Medicine (Baltimore). 2026 May 12;104(49):e46145. doi: 10.1097/MD.0000000000046145 (PMC12688818; doi:10.1097/MD.0000000000046145)
Supplement: Supplementary file 1 [file medi-104-e46145-s001.docx]

Table 1S. 2 × 2 contingency table of disproportionality method.

| Item | Target adverse events reported | Other adverse events reported | Total |
| --- | --- | --- | --- |
| Reports with the target drug | a | b | a+b |
| All other drugs | c | d | c+d |
| Total | a+c | b+d | a+b+c+d |

Note: a, b, c, d, indicated the number of reports that meet the specified conditions in the table.

Table 2S. The principles of disproportionate measurement and the criteria for signal detection.

| Method | Calculation formula | ﻿Criteria |
| --- | --- | --- |
| ROR | $ROR=\frac{a / c}{b / d}$ | a ≥ 3  ROR ≥ 1  95%CI (lower limit) > 1 |
|  | $SE(lnROR)=\sqrt{\frac{1}{a}+\frac{1}{b}+\frac{1}{c}+\frac{1}{d}}$ |  |
|  | $95\%CI= e^{\ln\left( ROR \right)\pm1.96se}$ |  |
| PRR | $PRR=\frac{a / (a+b)}{c / (c+d)}$ | a ≥ 3  PRR ≥ 2  χ^2^≥4 |
|  | $SE(lnPRR)=\sqrt{\frac{1}{a}-\frac{1}{a+b}+\frac{1}{c}-\frac{1}{c+d}}$ |  |
|  | χ^2^=[(ad-bc)^2](a+b+c+d)/[(a+b)(c+d)(a+c)(b+d)] |  |
| BCPNN | $IC=\log_{2}\frac{p(x, y)}{p\left( x \right)p(y)}= {log}_{2}\frac{a(a+b+c+d)}{(a+b)(a+c)}$ | IC025>0 |
|  | $E(IC)=\log_{2}\frac{(a+\gamma11)(a+b+c+d+\alpha)(a+b+c+d+\beta)}{\left( a+b+c+d+\gamma\right)(a+b+\alpha1)(a+c+\beta1)}$ |  |
|  | $V\left( \mathrm{IC} \right)=\frac{1}{{(ln2)}^{2}}[\frac{\left( a+b+c+d \right)-a+\gamma-\gamma11}{\left( a+\gamma11 \right)\left( 1+a+b+c+d+\gamma\right)}+\frac{\left( a+b+c+d \right)-\left( a+b \right)+a-\alpha1}{\left( a+b+\alpha1 \right)\left( 1+a+b+c+d+\alpha\right)}+\frac{\left( a+b+c+d+\alpha\right)-\left( a+c \right)+\beta-\beta1}{\left( a+b+\beta1 \right)\left( 1+a+b+c+d+\beta\right)}]$ |  |
|  | $\gamma=\gamma11\frac{(a+b+c+d+\alpha)(a+b+c+d+\beta)}{\left( a+b+\alpha1 \right)(a+c+\beta1)}$ |  |
|  | $IC-2SD=E\left( \mathrm{IC} \right)-2 \sqrt{V(IC)}$ |  |
|  | $\alpha1=\beta1=1:\alpha=\beta=2:\gamma11=1$ |  |
| EBGM | $EBGM=\frac{a(a+b+c+d)}{\left( a+c \right)(a+b)}$ | EBGM05>2 |
|  | $SE(lnEBGM)=\sqrt{\frac{1}{a}+\frac{1}{b}+\frac{1}{c}+\frac{1}{d}}$ |  |
|  | $95\%CI= e^{\ln\left( EBGM \right)\pm1.96se}$ |  |

Abbreviations: BCPNN, bayesian confidence propagation neural network; CI, confidence interval; IC, information component; ROR, reporting odds ratio.

Table 3S. Top 50 Signal Detection Based on PT Level Ranked by ROR.

| PTs | N | ROR (95%Cl) | PRR (χ²) | EBGM (EBGM05) | IC (IC025) |
| --- | --- | --- | --- | --- | --- |
| Spinal anaesthesia | 6 | 1269.14 (550.19 - 2927.56) | 1267.16 (6967.04) | 1163.09 (577.94) | 10.18 (9.04) |
| Therapeutic product effect prolonged | 23 | 1029.59 (673.52 - 1573.91) | 1023.42 (21908.30) | 954.46 (669.17) | 9.9 (9.29) |
| Horner's syndrome | 32 | 1016.94 (709.48 - 1457.64) | 1008.46 (30064.28) | 941.43 (696.56) | 9.88 (9.36) |
| Harlequin syndrome | 4 | 994.01 (360.49 - 2740.92) | 992.98 (3703.99) | 927.93 (397.13) | 9.86 (8.52) |
| Macular ischaemia | 7 | 848.12 (395.33 - 1819.53) | 846.58 (5578.31) | 798.84 (421.78) | 9.64 (8.59) |
| Phrenic nerve paralysis | 11 | 843.76 (458.87 - 1551.5) | 841.35 (8714.68) | 794.18 (477.07) | 9.63 (8.77) |
| Nerve root injury | 4 | 798.01 (291.31 - 2186.04) | 797.18 (3011.08) | 754.72 (324.78) | 9.56 (8.23) |
| Femoral nerve injury | 3 | 772.42 (241.54 - 2470.1) | 771.81 (2189.99) | 731.94 (276.72) | 9.52 (8.02) |
| Anterior spinal artery syndrome | 4 | 745.51 (272.63 - 2038.61) | 744.73 (2822.39) | 707.55 (304.93) | 9.47 (8.14) |
| Maternal exposure during delivery | 31 | 650.32 (453.10 - 933.39) | 645.07 (19066.27) | 616.99 (456.00) | 9.27 (8.75) |
| Delayed recovery from anaesthesia | 26 | 458.42 (309.81 - 678.31) | 455.32 (11419.09) | 441.16 (317.84) | 8.79 (8.22) |
| Local anaesthetic systemic toxicity | 18 | 454.52 (283.97 - 727.51) | 452.4 (7856.07) | 438.41 (295.77) | 8.78 (8.10) |
| Diaphragmatic paralysis | 14 | 373.03 (219.21 - 634.77) | 371.67 (5042.97) | 362.18 (232.14) | 8.5 (7.74) |
| Epidural haemorrhage | 3 | 363.1 (115.38 - 1142.69) | 362.82 (1055.40) | 353.77 (135.55) | 8.47 (7.00) |
| Neuromuscular block prolonged | 8 | 334.62 (165.87 - 675.06) | 333.92 (2594.19) | 326.25 (181.35) | 8.35 (7.37) |
| Spinal cord ischaemia | 3 | 303.45 (96.65 - 952.72) | 303.21 (884.69) | 296.87 (113.98) | 8.21 (6.75) |
| Anaesthetic complication | 50 | 298.05 (224.84 - 395.09) | 294.18 (14312.13) | 288.21 (227.66) | 8.17 (7.76) |
| Cauda equina syndrome | 12 | 251.6 (142.05 - 445.63) | 250.81 (2933.81) | 246.46 (152.76) | 7.95 (7.14) |
| Suspected product quality issue | 21 | 228.25 (148.14 - 351.68) | 227 (4650.68) | 223.44 (155.62) | 7.8 (7.18) |
| Neuromuscular blockade | 3 | 216.75 (69.27 - 678.18) | 216.58 (634.05) | 213.33 (82.14) | 7.74 (6.28) |
| Laryngeal dyspnoea | 3 | 198.52 (63.49 - 620.69) | 198.36 (580.96) | 195.63 (75.37) | 7.61 (6.16) |
| Bradycardia foetal | 10 | 193.54 (103.61 - 361.52) | 193.04 (1884.78) | 190.46 (112.91) | 7.57 (6.70) |
| Peripheral nerve injury | 10 | 191.45 (102.5 - 357.6) | 190.96 (1864.49) | 188.43 (111.71) | 7.56 (6.68) |
| Sensorimotor disorder | 6 | 190.23 (84.95 - 425.97) | 189.93 (1112.70) | 187.43 (95.48) | 7.55 (6.45) |
| Anaesthetic complication neurological | 7 | 163.74 (77.68 - 345.18) | 163.45 (1117.28) | 161.59 (86.58) | 7.34 (6.31) |
| Amyotrophy | 4 | 151.49 (56.53 - 405.97) | 151.34 (591.05) | 149.75 (65.64) | 7.23 (5.93) |
| Spinal cord infarction | 4 | 143.08 (53.41 - 383.3) | 142.93 (558.11) | 141.51 (62.04) | 7.14 (5.85) |
| Amaurosis | 7 | 141.55 (67.19 - 298.23) | 141.3 (965.50) | 139.91 (75.00) | 7.13 (6.10) |
| Arachnoiditis | 6 | 133.28 (59.61 - 297.97) | 133.07 (779.15) | 131.84 (67.25) | 7.04 (5.95) |
| Clonic convulsion | 5 | 107.33 (44.50 - 258.88) | 107.2 (522.08) | 106.4 (50.93) | 6.73 (5.55) |
| Paraparesis | 11 | 95.64 (52.82 - 173.2) | 95.37 (1020.37) | 94.74 (57.64) | 6.57 (5.73) |
| Chondrolysis | 3 | 91.16 (29.28 - 283.82) | 91.09 (265.61) | 90.52 (35.00) | 6.5 (5.05) |
| Nerve block | 3 | 85.31 (27.41 - 265.52) | 85.24 (248.26) | 84.74 (32.77) | 6.4 (4.96) |
| Brain stem syndrome | 4 | 84.31 (31.54 - 225.42) | 84.23 (327.01) | 83.73 (36.77) | 6.39 (5.09) |
| Paraplegia | 15 | 82.11 (49.38 - 136.53) | 81.79 (1190.23) | 81.33 (53.14) | 6.35 (5.62) |
| Diaphragmatic disorder | 4 | 79.35 (29.69 - 212.12) | 79.27 (307.42) | 78.84 (34.63) | 6.3 (5.01) |
| Diplegia | 10 | 77.78 (41.74 - 144.92) | 77.58 (751.80) | 77.16 (45.84) | 6.27 (5.40) |
| Post lumbar puncture syndrome | 5 | 73.49 (30.50 - 177.06) | 73.39 (355.19) | 73.02 (34.98) | 6.19 (5.01) |
| Allodynia | 4 | 70.03 (26.21 - 187.15) | 69.96 (270.57) | 69.62 (30.59) | 6.12 (4.83) |
| Monoplegia | 18 | 64.18 (40.35 - 102.08) | 63.88 (1109.24) | 63.6 (43.13) | 5.99 (5.33) |
| Bundle branch block | 4 | 63.88 (23.91 - 170.66) | 63.81 (246.20) | 63.53 (27.92) | 5.99 (4.69) |
| Tonic clonic movements | 5 | 60.6 (25.16 - 145.95) | 60.52 (291.45) | 60.27 (28.89) | 5.91 (4.73) |
| Postictal state | 4 | 59.64 (22.33 - 159.32) | 59.58 (229.42) | 59.33 (26.08) | 5.89 (4.60) |
| Vasoconstriction | 3 | 57.64 (18.54 - 179.22) | 57.60 (166.17) | 57.37 (22.21) | 5.84 (4.39) |
| Product packaging confusion | 9 | 57.26 (29.73 - 110.29) | 57.13 (494.38) | 56.91 (32.88) | 5.83 (4.91) |
| Axonal neuropathy | 3 | 57.02 (18.34 - 177.29) | 56.98 (164.33) | 56.75 (21.97) | 5.83 (4.38) |
| Clonus | 9 | 51.12 (26.55 - 98.44) | 51 (439.62) | 50.82 (29.37) | 5.67 (4.75) |
| Unmasking of previously unidentified disease | 3 | 47.89 (15.41 - 148.85) | 47.86 (137.17) | 47.7 (18.47) | 5.58 (4.13) |
| Paresis | 7 | 47.45 (22.58 - 99.73) | 47.37 (316.69) | 47.21 (25.36) | 5.56 (4.54) |
| Radiculopathy | 8 | 46.89 (23.41 - 93.94) | 46.8 (357.37) | 46.64 (26.08) | 5.54 (4.58) |

Abbreviation: OR, reporting odds ratio; CI, confidence interval; PRR, proportional reporting ratio; χ2, chi-squared; EBGM, empirical Bayesian geometric mean; EBGM, 05, the lower limit of 95% CI, of EBGM; IC, information component; IC025, the lower limit of 95% CI, of the IC. N: Case numbers.

Extremely high ROR values (such as >1000) are usually associated with rare events and a small number of case reports, and should be interpreted with caution.

Table 4S. The PT level signal intensity among the female population (top 50 ranked by ROR)

| PTs | N | ROR (95%Cl) | PRR (χ²) | EBGM (EBGM05) | IC (IC025) |
| --- | --- | --- | --- | --- | --- |
| Spinal anaesthesia | 6 | 2065.08 ( 879.04 - 4851.38 ) | 2058.96 ( 10860.76 ) | 1812 ( 886.73 ) | 10.82 ( 9.66 ) |
| Horner's syndrome | 28 | 1721.69 ( 1162.03 - 2550.87 ) | 1697.88 ( 42685.21 ) | 1526.36 ( 1098.49 ) | 10.58 ( 10.01 ) |
| Nerve root injury | 4 | 1186.58 ( 428.46 - 3286.15 ) | 1184.24 ( 4385.04 ) | 1098.18 ( 468.29 ) | 10.1 ( 8.75 ) |
| Anterior spinal artery syndrome | 3 | 855.93 ( 267.25 - 2741.32 ) | 854.66 ( 2420.96 ) | 808.93 ( 305.44 ) | 9.66 ( 8.17 ) |
| Epidural haemorrhage | 3 | 824.8 ( 257.82 - 2638.65 ) | 823.58 ( 2337.27 ) | 781.04 ( 295.18 ) | 9.61 ( 8.12 ) |
| Maternal exposure during delivery | 30 | 767.57 ( 530.55 - 1110.48 ) | 756.21 ( 21547.67 ) | 720.19 ( 528.74 ) | 9.49 ( 8.96 ) |
| Anaesthetic complication neurological | 6 | 644.42 ( 284.34 - 1460.49 ) | 642.51 ( 3686.24 ) | 616.33 ( 310.81 ) | 9.27 ( 8.15 ) |
| Diaphragmatic paralysis | 9 | 601.31 ( 308.44 - 1172.27 ) | 598.64 ( 5165.03 ) | 575.85 ( 329.39 ) | 9.17 ( 8.24 ) |
| Laryngeal dyspnoea | 3 | 391.07 ( 124.2 - 1231.34 ) | 390.49 ( 1136.11 ) | 380.67 ( 145.8 ) | 8.57 ( 7.10 ) |
| Cauda equina syndrome | 9 | 388.88 ( 200.37 - 754.73 ) | 387.15 ( 3379.79 ) | 377.5 ( 216.75 ) | 8.56 ( 7.63 ) |
| Anaesthetic complication | 32 | 351.15 ( 246.67 - 499.89 ) | 345.61 ( 10750.19 ) | 337.9 ( 251.45 ) | 8.4 ( 7.89 ) |
| Peripheral nerve injury | 10 | 349.63 ( 186.5 - 655.43 ) | 347.9 ( 3381.21 ) | 340.09 ( 201.02 ) | 8.41 ( 7.53 ) |
| Neuromuscular block prolonged | 3 | 273.28 ( 87.17 - 856.72 ) | 272.87 ( 798.21 ) | 268.05 ( 103.04 ) | 8.07 ( 6.61 ) |
| Local anaesthetic systemic toxicity | 5 | 249.77 ( 103.1 - 605.09 ) | 249.16 ( 1215.76 ) | 245.13 ( 116.91 ) | 7.94 ( 6.75 ) |
| Arachnoiditis | 5 | 235.77 ( 97.36 - 570.93 ) | 235.19 ( 1148.09 ) | 231.6 ( 110.50 ) | 7.86 ( 6.67 ) |
| Brain stem syndrome | 4 | 198.41 ( 73.92 - 532.57 ) | 198.02 ( 773.96 ) | 195.47 ( 85.56 ) | 7.61 ( 6.31 ) |
| Clonic convulsion | 4 | 182.82 ( 68.15 - 490.49 ) | 182.47 ( 713.27 ) | 180.3 ( 78.95 ) | 7.49 ( 6.19 ) |
| Bradycardia foetal | 3 | 165.56 ( 53.02 - 516.96 ) | 165.32 ( 484.67 ) | 163.54 ( 63.07 ) | 7.35 ( 5.90 ) |
| Paraplegia | 12 | 151.38 ( 85.58 - 267.76 ) | 150.49 ( 1764.43 ) | 149.01 ( 92.47 ) | 7.22 ( 6.41 ) |
| Amaurosis | 3 | 148.25 ( 47.51 - 462.6 ) | 148.03 ( 433.86 ) | 146.6 ( 56.57 ) | 7.2 ( 5.74 ) |
| Delayed recovery from anaesthesia | 4 | 139.44 ( 52.05 - 373.56 ) | 139.16 ( 543.67 ) | 137.9 ( 60.46 ) | 7.11 ( 5.81 ) |
| Post lumbar puncture syndrome | 5 | 138.61 ( 57.4 - 334.72 ) | 138.27 ( 675.21 ) | 137.02 ( 65.53 ) | 7.1 ( 5.91 ) |
| Unmasking of previously unidentified disease | 3 | 138.3 ( 44.34 - 431.41 ) | 138.1 ( 404.63 ) | 136.86 ( 52.83 ) | 7.1 ( 5.64 ) |
| Allodynia | 4 | 123.25 ( 46.03 - 330.02 ) | 123.01 ( 480.15 ) | 122.02 ( 53.52 ) | 6.93 ( 5.63 ) |
| Product packaging confusion | 4 | 114.61 ( 42.81 - 306.81 ) | 114.39 ( 446.21 ) | 113.53 ( 49.81 ) | 6.83 ( 5.53 ) |
| Diplegia | 6 | 90.05 ( 40.31 - 201.16 ) | 89.79 ( 523.69 ) | 89.26 ( 45.56 ) | 6.48 ( 5.38 ) |
| Paraparesis | 5 | 87.09 ( 36.12 - 209.99 ) | 86.88 ( 422.02 ) | 86.38 ( 41.36 ) | 6.43 ( 5.25 ) |
| Monoplegia | 13 | 86.26 ( 49.92 - 149.05 ) | 85.71 ( 1082.38 ) | 85.24 ( 53.94 ) | 6.41 ( 5.64 ) |
| Clonus | 7 | 83.05 ( 39.46 - 174.79 ) | 82.77 ( 562.39 ) | 82.32 ( 44.17 ) | 6.36 ( 5.34 ) |
| Myelitis transverse | 3 | 68.01 ( 21.86 - 211.59 ) | 67.91 ( 196.9 ) | 67.61 ( 26.16 ) | 6.08 ( 4.63 ) |
| Quadriparesis | 3 | 65.65 ( 21.1 - 204.22 ) | 65.55 ( 189.88 ) | 65.27 ( 25.25 ) | 6.03 ( 4.58 ) |
| Areflexia | 5 | 59.4 ( 24.66 - 143.12 ) | 59.26 ( 285.27 ) | 59.03 ( 28.28 ) | 5.88 ( 4.70 ) |
| Neurotoxicity | 20 | 54.83 ( 35.27 - 85.23 ) | 54.29 ( 1042.68 ) | 54.1 ( 37.40 ) | 5.76 ( 5.12 ) |
| Paresis | 4 | 54.81 ( 20.52 - 146.45 ) | 54.71 ( 210.15 ) | 54.51 ( 23.95 ) | 5.77 ( 4.47 ) |
| Cerebrospinal fluid leakage | 3 | 53.81 ( 17.31 - 167.33 ) | 53.73 ( 154.71 ) | 53.55 ( 20.72 ) | 5.74 ( 4.3 ) |
| Stridor | 5 | 53.11 ( 22.05 - 127.93 ) | 52.98 ( 254.11 ) | 52.8 ( 25.30 ) | 5.72 ( 4.54 ) |
| Dysaesthesia | 5 | 48.36 ( 20.08 - 116.47 ) | 48.24 ( 230.58 ) | 48.09 ( 23.05 ) | 5.59 ( 4.41 ) |
| Atrioventricular block second degree | 4 | 45.98 ( 17.22 - 122.82 ) | 45.89 ( 175.14 ) | 45.76 ( 20.11 ) | 5.52 ( 4.22 ) |
| Generalised tonic-clonic seizure | 20 | 43.87 ( 28.22 - 68.19 ) | 43.44 ( 827.15 ) | 43.32 ( 29.95 ) | 5.44 ( 4.80 ) |
| Meningitis aseptic | 6 | 42.16 ( 18.90 - 94.06 ) | 42.04 ( 239.73 ) | 41.93 ( 21.42 ) | 5.39 ( 4.30 ) |
| Oliguria | 7 | 38.48 ( 18.30 - 80.90 ) | 38.35 ( 254.01 ) | 38.26 ( 20.54 ) | 5.26 ( 4.23 ) |
| Motor dysfunction | 12 | 36.34 ( 20.59 - 64.14 ) | 36.13 ( 408.97 ) | 36.05 ( 22.41 ) | 5.17 ( 4.37 ) |
| Facial paresis | 5 | 36.19 ( 15.03 - 87.14 ) | 36.1 ( 170.27 ) | 36.02 ( 17.27 ) | 5.17 ( 3.99 ) |
| Apnoea | 8 | 35.66 ( 17.80 - 71.47 ) | 35.53 ( 267.84 ) | 35.45 ( 19.81 ) | 5.15 ( 4.18 ) |
| Sensory loss | 10 | 35.46 ( 19.03 - 66.05 ) | 35.29 ( 332.42 ) | 35.21 ( 20.92 ) | 5.14 ( 4.26 ) |
| Deafness neurosensory | 3 | 34.21 ( 11.01 - 106.3 ) | 34.16 ( 96.36 ) | 34.09 ( 13.20 ) | 5.09 ( 3.64 ) |
| Radiculopathy | 3 | 30.99 ( 9.97 - 96.27 ) | 30.94 ( 86.75 ) | 30.88 ( 11.96 ) | 4.95 ( 3.50 ) |
| Hypovolaemia | 5 | 30.07 ( 12.49 - 72.38 ) | 29.99 ( 139.87 ) | 29.94 ( 14.35 ) | 4.9 ( 3.72 ) |
| Ventricular fibrillation | 7 | 26.09 ( 12.42 - 54.84 ) | 26.01 ( 168.05 ) | 25.96 ( 13.95 ) | 4.7 ( 3.68 ) |
| Anaphylactic shock | 20 | 24.09 ( 15.51 - 37.44 ) | 23.87 ( 437.65 ) | 23.83 ( 16.48 ) | 4.57 ( 3.94 ) |

Abbreviation: ROR, reporting odds ratio; CI, confidence interval; PRR, proportional reporting ratio; χ2, chi-squared; EBGM, empirical Bayesian geometric mean; EBGM, 05, the lower limit of 95% CI, of EBGM; IC, information component; IC025, the lower limit of 95% CI, of the IC. N: Case numbers.

Extremely high ROR values (such as >1000) are usually associated with rare events and a small number of case reports, and should be interpreted with caution.

Table 5S. The PT level signal intensity among the male population (top 50 ranked by ROR)

| PT | N | ROR (95%Cl) | PRR (χ²) | EBGM (EBGM05) | IC (IC025) |
| --- | --- | --- | --- | --- | --- |
| Phrenic nerve paralysis | 9 | 1354.79 ( 682.41 - 2689.66 ) | 1345.36 ( 11044.04 ) | 1229.02 ( 692.40 ) | 10.26 ( 9.31 ) |
| Local anaesthetic systemic toxicity | 10 | 935.4 ( 492.21 - 1777.67 ) | 928.17 ( 8693.60 ) | 871.29 ( 509.14 ) | 9.77 ( 8.86 ) |
| Spinal cord ischaemia | 3 | 593.08 ( 186.63 - 1884.78 ) | 591.71 ( 1698.38 ) | 568.08 ( 215.90 ) | 9.15 ( 7.67 ) |
| Sensorimotor disorder | 4 | 331.28 ( 122.75 - 894.08 ) | 330.26 ( 1283.21 ) | 322.77 ( 140.64 ) | 8.33 ( 7.02 ) |
| Anaesthetic complication | 16 | 326.79 ( 198.49 - 538.01 ) | 322.75 ( 5018.21 ) | 315.6 ( 207.95 ) | 8.3 ( 7.59 ) |
| Neuromuscular block prolonged | 3 | 325.97 ( 103.65 - 1025.15 ) | 325.21 ( 947.95 ) | 317.96 ( 121.90 ) | 8.31 ( 6.85 ) |
| Diaphragmatic paralysis | 5 | 275.21 ( 113.40 - 667.93 ) | 274.15 ( 1335.02 ) | 268.98 ( 128.09 ) | 8.07 ( 6.88 ) |
| Delayed recovery from anaesthesia | 5 | 247.5 ( 102.06 - 600.17 ) | 246.55 ( 1201.90 ) | 242.36 ( 115.49 ) | 7.92 ( 6.73 ) |
| Spinal cord infarction | 3 | 241.25 ( 76.97 - 756.20 ) | 240.7 ( 704.17 ) | 236.70 ( 91.00 ) | 7.89 ( 6.43 ) |
| Product packaging confusion | 5 | 194.22 ( 80.22 - 470.21 ) | 193.47 ( 944.55 ) | 190.89 ( 91.09 ) | 7.58 ( 6.39 ) |
| Diaphragmatic disorder | 4 | 191.85 ( 71.42 - 515.33 ) | 191.26 ( 747.01 ) | 188.73 ( 82.56 ) | 7.56 ( 6.26 ) |
| Amaurosis | 4 | 171.11 ( 63.75 - 459.29 ) | 170.58 ( 666.36 ) | 168.57 ( 73.79 ) | 7.40 ( 6.09 ) |
| Cauda equina syndrome | 3 | 161.14 ( 51.57 - 503.49 ) | 160.77 ( 470.99 ) | 158.98 ( 61.28 ) | 7.31 ( 5.86 ) |
| Bundle branch block | 4 | 131.29 ( 48.98 - 351.92 ) | 130.89 ( 510.87 ) | 129.7 ( 56.84 ) | 7.02 ( 5.72 ) |
| Paraparesis | 6 | 129.89 ( 58.04 - 290.73 ) | 129.3 ( 756.96 ) | 128.14 ( 65.3 ) | 7.00 ( 5.90 ) |
| Tonic clonic movements | 4 | 117.24 ( 43.76 - 314.12 ) | 116.88 ( 455.82 ) | 115.93 ( 50.83 ) | 6.86 ( 5.56 ) |
| Radiculopathy | 5 | 91.03 ( 37.72 - 219.69 ) | 90.68 ( 440.68 ) | 90.11 ( 43.12 ) | 6.49 ( 5.31 ) |
| Diplegia | 4 | 80.36 ( 30.03 - 215.05 ) | 80.12 ( 310.78 ) | 79.67 ( 34.97 ) | 6.32 ( 5.02 ) |
| Wound infection staphylococcal | 3 | 70.46 ( 22.63 - 219.38 ) | 70.3 ( 203.95 ) | 69.96 ( 27.05 ) | 6.13 ( 4.68 ) |
| Arteriospasm coronary | 7 | 63.94 ( 30.37 - 134.62 ) | 63.6 ( 429.43 ) | 63.32 ( 33.96 ) | 5.98 ( 4.96 ) |
| Injection site infection | 4 | 56.75 ( 21.23 - 151.73 ) | 56.58 ( 217.53 ) | 56.36 ( 24.75 ) | 5.82 ( 4.52 ) |
| Electrocardiogram qrs complex prolonged | 6 | 52.67 ( 23.59 - 117.64 ) | 52.43 ( 301.63 ) | 52.25 ( 26.67 ) | 5.71 ( 4.61 ) |
| Stress cardiomyopathy | 3 | 51.26 ( 16.48 - 159.47 ) | 51.14 ( 146.97 ) | 50.96 ( 19.72 ) | 5.67 ( 4.22 ) |
| Paresis | 3 | 50.06 ( 16.09 - 155.73 ) | 49.94 ( 143.40 ) | 49.77 ( 19.26 ) | 5.64 ( 4.19 ) |
| Nosocomial infection | 3 | 49.31 ( 15.85 - 153.39 ) | 49.2 ( 141.16 ) | 49.03 ( 18.97 ) | 5.62 ( 4.17 ) |
| Methaemoglobinaemia | 3 | 46.72 ( 15.02 - 145.31 ) | 46.61 ( 133.47 ) | 46.46 ( 17.98 ) | 5.54 ( 4.09 ) |
| Retinal artery occlusion | 3 | 45.52 ( 14.64 - 141.59 ) | 45.42 ( 129.91 ) | 45.28 ( 17.52 ) | 5.5 ( 4.05 ) |
| Peroneal nerve palsy | 5 | 42.99 ( 17.84 - 103.59 ) | 42.83 ( 203.65 ) | 42.7 ( 20.46 ) | 5.42 ( 4.23 ) |
| Tachyarrhythmia | 3 | 42.11 ( 13.54 - 130.96 ) | 42.01 ( 119.77 ) | 41.89 ( 16.21 ) | 5.39 ( 3.94 ) |
| Monoplegia | 4 | 40.41 ( 15.12 - 107.98 ) | 40.29 ( 152.82 ) | 40.18 ( 17.65 ) | 5.33 ( 4.03 ) |
| Procedural complication | 8 | 39.95 ( 19.92 - 80.14 ) | 39.71 ( 301.08 ) | 39.6 ( 22.12 ) | 5.31 ( 4.34 ) |
| Ventricular fibrillation | 14 | 38.6 ( 22.78 - 65.40 ) | 38.19 ( 505.80 ) | 38.09 ( 24.50 ) | 5.25 ( 4.50 ) |
| Paraplegia | 3 | 36.46 ( 11.73 - 113.37 ) | 36.38 ( 102.97 ) | 36.29 ( 14.05 ) | 5.18 ( 3.73 ) |
| Anaphylactic shock | 18 | 33.23 ( 20.86 - 52.95 ) | 32.78 ( 553.62 ) | 32.71 ( 22.15 ) | 5.03 ( 4.36 ) |
| Amylase increased | 3 | 30.96 ( 9.96 - 96.25 ) | 30.89 ( 86.60 ) | 30.83 ( 11.94 ) | 4.95 ( 3.5 ) |
| Sinus tachycardia | 10 | 29.06 ( 15.59 - 54.18 ) | 28.85 ( 268.34 ) | 28.79 ( 17.10 ) | 4.85 ( 3.97 ) |
| Wrong product administered | 3 | 24.81 ( 7.98 - 77.10 ) | 24.75 ( 68.27 ) | 24.71 ( 9.57 ) | 4.63 ( 3.18 ) |
| Motor dysfunction | 6 | 24.36 ( 10.91 - 54.35 ) | 24.25 ( 133.53 ) | 24.21 ( 12.37 ) | 4.6 ( 3.50 ) |
| Post procedural complication | 10 | 21.91 ( 11.75 - 40.83 ) | 21.74 ( 197.67 ) | 21.71 ( 12.9 ) | 4.44 ( 3.57 ) |
| Supraventricular tachycardia | 5 | 21.53 ( 8.94 - 51.85 ) | 21.45 ( 97.36 ) | 21.42 ( 10.27 ) | 4.42 ( 3.24 ) |
| Neurotoxicity | 9 | 21.22 ( 11.01 - 40.89 ) | 21.08 ( 171.92 ) | 21.05 ( 12.16 ) | 4.4 ( 3.48 ) |
| Cardiotoxicity | 3 | 20.88 ( 6.72 - 64.88 ) | 20.83 ( 56.57 ) | 20.8 ( 8.06 ) | 4.38 ( 2.93 ) |
| Acute pulmonary oedema | 3 | 19.86 ( 6.39 - 61.71 ) | 19.82 ( 53.53 ) | 19.79 ( 7.66 ) | 4.31 ( 2.86 ) |
| Apnoea | 4 | 18.4 ( 6.89 - 49.12 ) | 18.34 ( 65.51 ) | 18.32 ( 8.05 ) | 4.2 ( 2.90 ) |
| Nerve injury | 4 | 17.43 ( 6.53 - 46.55 ) | 17.38 ( 61.69 ) | 17.36 ( 7.63 ) | 4.12 ( 2.82 ) |
| Sensory disturbance | 5 | 16.72 ( 6.94 - 40.26 ) | 16.66 ( 73.53 ) | 16.64 ( 7.98 ) | 4.06 ( 2.88 ) |
| Posterior reversible encephalopathy syndrome | 3 | 16.45 ( 5.30 - 51.12 ) | 16.42 ( 43.39 ) | 16.4 ( 6.35 ) | 4.04 ( 2.59 ) |
| Paraesthesia oral | 3 | 16.45 ( 5.29 - 51.10 ) | 16.41 ( 43.37 ) | 16.39 ( 6.35 ) | 4.04 ( 2.59 ) |
| Generalised tonic-clonic seizure | 6 | 15.73 ( 7.05 - 35.10 ) | 15.67 ( 82.31 ) | 15.65 ( 8.00 ) | 3.97 ( 2.87 ) |
| Procedural pain | 5 | 15.67 ( 6.51 - 37.72 ) | 15.61 ( 68.31 ) | 15.59 ( 7.47 ) | 3.96 ( 2.78 ) |

Abbreviation: ROR, reporting odds ratio; CI, confidence interval; PRR, proportional reporting ratio; χ2, chi-squared; EBGM, empirical Bayesian geometric mean; EBGM, 05, the lower limit of 95% CI, of EBGM; IC, information component; IC025, the lower limit of 95% CI, of the IC. N: Case numbers.

Extremely high ROR values (such as >1000) are usually associated with rare events and a small number of case reports, and should be interpreted with caution.
